# Effects of Shoe Midfoot Bending Stiffness on Multi-Segment Foot Kinematics and Ground Reaction Force during Heel-Toe Running

**DOI:** 10.3390/bioengineering9100520

**Published:** 2022-10-02

**Authors:** Ruiya Ma, Wing-Kai Lam, Rui Ding, Fan Yang, Feng Qu

**Affiliations:** 1 Biomechanics Laboratory, School of Sport Science, Beijing Sport University, Beijing 100084, China; 2 Sports Information and External Affairs Center, Hong Kong Sports Institute, Hong Kong 999077, China; 3 Li Ning Sports Science Research Center, Li Ning (China) Sports Goods Company Limited, Beijing 101111, China; 4 School of Sports Science, Lingnan Normal University, Zhanjiang 524048, China

**Keywords:** midsole modification, overground running, foot kinematics, running injuries, footwear

## Abstract

We investigated how midfoot stiffness of running shoes influences foot segment kinematics and ground reaction force (GRF) during heel-toe running. Nineteen male rearfoot strike runners performed overground heel-toe running at 3.3 m/s when wearing shoes with different midfoot bending stiffnesses (low, medium, and high) in a randomized order. A synchronized motion capture system (200 Hz) and force plate (1000 Hz) were used to collect the foot-marker trajectories and GRF data. Foot kinematics, including rearfoot-lab, midfoot-rearfoot, forefoot-rearfoot, and forefoot-midfoot interactions, and kinetics, including GRF characteristics, were analyzed. Our results indicated that high midfoot stiffness shoes reduced the forefoot-rearfoot range of motion (mean ± SD; high stiffness, 7.8 ± 2.0°, low stiffness, 8.7 ± 2.1°; *p* < 0.05) and forefoot-midfoot range of motion (mean ± SD; high stiffness, 4.2 ± 1.1°, medium stiffness, 4.6 ± 0.9°; *p* < 0.05) in the frontal plane. No differences were found in the GRF characteristics among the shoe conditions. These findings suggest that an increase in midsole stiffness only in the midfoot region can reduce intersegmental foot medial-lateral movements during the stance phase of running. This may further decrease the tension of the foot muscles and tendons during prolonged exercises.

## 1. Introduction

Running is one of the most popular activities, and footwear is commonly used during running exercises, except for barefoot running. Footwear constructions and features such as midsole hardness, midsole thickness, heel-toe drop, heel counter, longitudinal bending stiffness, and torsional stiffness would significantly alter foot position and joint loading, which is aimed at improving running performance and preventing running injuries [1,2]. Footwear scientists have explored the effect of isolated running shoe features that have the potential to optimize shoe characteristics and performance for decades.

The longitudinal bending stiffness is described as shoe rotation around the medial-lateral axis and is considered to be one of the key features in running shoe development. This can be optimized by incorporating higher-density/thicker midsoles, carbon fiber/thermoplastic polyurethane (TPU) plates, or flex grooves [1]. The foot is frequently considered a rigid segment in running biomechanical research [3,4,5,6]. However, intersegmental motion cannot be neglected because the degree of motion is comparable to ankle motion during walking [7,8,9]. Studying shoe bending stiffness would influence intersegmental foot kinematics and can assist in understanding the intrinsic structural function of the foot during movements.

Previous studies examined the effect of shoe bending stiffness on arch movement. Cigoja et al. [10] suggested that the absolute peak change in the arch angle was about an average of 1.6° smaller in the shoes with a carbon plate than in the control shoe condition. Renan et al. [11] found that shoes with drilled holes in the forefoot increased the forefoot-rearfoot (forefoot with respect to the rearfoot) maximum dorsiflexion angle and the sagittal range of motion during walking. Both studies indicated that increased shoe midsole stiffness is related to decreased arch deformation.

Moreover, Renan et al. [11] found that shoes with lower forefoot stiffness would lead to an increased forefoot-rearfoot maximum inversion angle during walking. The forefoot-rearfoot movement in the frontal plane is described as a foot torsional motion [12], which can be restricted when the shoe torsional stiffness increases [13,14]. Arndt et al. [15] demonstrated that shoe bending and torsional stiffness decreased when a deep groove was cut underneath the midfoot region of the shoe sole. However, no significant changes were found in the intersegmental foot kinematics between the cut and uncut shoes. Since the forefoot-rearfoot motion was reported to be larger during running than in walking [16], the modification of shoe midfoot stiffness might impact running to a greater extent than walking.

Stiff shoes with carbon fiber plates have been reported to systematically reduce the metatarsophalangeal (MTP) maximum dorsiflexion and plantar flexion velocities [17] and peak arch flexion velocity [10] during the propulsion phase of running, which indicates that increased stiffness would reduce foot propulsion velocity. The midfoot stiffness may produce similar results, as the midfoot connects with the rearfoot and forefoot and contributes to the foot transition from heel contact to toe-off.

Shoe bending stiffness also influences the ground reaction force (GRF) data. Shoes with carbon fiber insoles decreased the peak GRF in the anterior-posterior direction. They increased the stance duration while the anterior-posterior GRF impulse and peak vertical GRF remained unchanged [18]. Flores et al. [19] demonstrated that an increased shoe bending stiffness decreases the GRF after the vertical and anterior propulsion peaks during running using statistical parametric mapping. It is unknown whether increased midfoot bending stiffness could decrease the GRF during the stance phase of running.

The effect and underlying mechanism of shoe midfoot stiffness on intersegmental foot movement and GRF during running are still questionable. Hence, the objective of this study was to examine the effect of shoe midfoot bending stiffness on intersegmental foot kinematics using a multi-segment foot model and GRF characteristics. By gradually increasing midfoot stiffness, we hypothesized that stiffer midsoles would exhibit smaller intersegmental motion in the sagittal and frontal planes, slower propulsion velocity, and smaller GRF during running.

## 2. Materials and Methods

### 2.1. Participants

A sample size of 12 was calculated based on an analysis of variance (ANOVA: repeated measures, within factors) to provide sufficient statistical power (0.8) to detect a large effect size (η_p_^2^ = 0.14, effect size f = 0.403) to examine the differences between the three tested conditions at 0.05 when the correlation among repeated measures was set at 0.5 using G*Power version 3.1.9.7 [20]. Nineteen male recreational runners (aged 22.3 ± 2.5 years, height 1.80 ± 0.04 m, mass 71.3 ± 5.8 kg) were recruited from a local sports university. To fit into the experimental shoes, the participants’ heel-toe length was between US 8.5 and 9.5 (The Brannock Device Company, Liverpool, NY, USA). Participants reported in a questionnaire that their average running experience and exposure were 7.6 ± 2.2 years and 15.7 ± 5.6 km/week, respectively. All participants were lower extremity injury-free for at least six months before the study, and they were right-leg dominant, which was determined by the leg they kicked a ball [21]. During data collection, any participant who showed a forefoot or midfoot running strike pattern characterized by the heel marker trajectory reaching its lowest point later than the forefoot/midfoot markers or only a simple peak found in the vertical GRF curve was excluded [22,23]. Each participant signed an informed consent form before the start of data collection. Ethics approval was granted by the Institutional Review Board of Beijing Sport University (IRB Number: 2021146H).

### 2.2. Footwear Conditions

**Figure 1:**
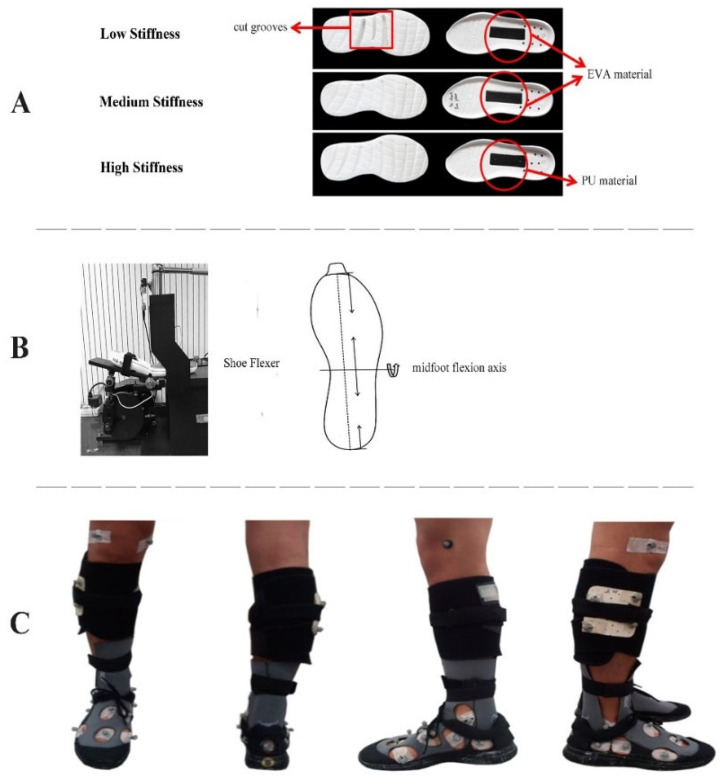
(**A**) Constructions of shoe conditions (low, medium, and high), with ethylene-vinyl acetate copolymer (EVA) or thermoplastic polyurethane (TPU) plate in the midfoot, (**B**) Bending axis of midfoot mechanical flexion test, and (**C**) Marker placement on the body with the subject shod.

Three shoe conditions with varying midfoot bending stiffness (high, medium, and low) were built from a neutral running shoe model with a midsole ethylene-vinyl acetate copolymer (EVA) hardness of Asker C 45 (LN ARJH001, Li Ning, Beijing, China). The medium stiffness shoe was identical to the original shoe model. The low stiffness shoe was modified by cutting with three 1 cm depth and 0.9–1.8 cm width transverse grooves at the midfoot region. In contrast, the high stiffness shoe was modified by replacing the original EVA midfoot plate with a stiffer TPU midfoot plate (Shore D 70). All three shoe conditions had the same size (9.5 cm × 2.5 cm × 0.2 cm) and location of the midfoot shank plate as per the manufacturer’s specification (Figure 1A). The longitudinal midfoot bending stiffness was quantified using a mechanical shoe flexion tester (Exeter Research, Brentwood, NH, USA) by bending across the midfoot flexion axis located 45% of the shoe length from the heel for each shoe (Figure 1B). Sixty consecutive flexion-extension cycles were performed and the stiffness between 10° and 25° of the 51st to 55th trials was averaged to denote the midfoot bending stiffness (modified according to the forefoot flexibility test standard: ASTM 911-85). The bending stiffness values for the low, medium, and high stiffness shoes were 0.069, 0.091, and 0.163 Nm/°, respectively.

There are different approaches to marker attachment for foot kinematics measurement: shoe surface markers, foot skin markers, and bone-mounted markers. Arnold and Bishop [24] reviewed articles and concluded that it is inappropriate to describe in-shoe foot motion with markers placed on the external shoe surface owing to the induced errors and lack of validity. However, the bone-mounted marker approach is not widely accepted because of its invasive preparation procedures [15]. Attaching markers directly to the skin by drilling holes in the shoes is an alternative approach for studying multi-segment foot motion [25,26]. Furthermore, to accurately assess the actual foot motion, Sterzing et al. [27] removed the original shoe upper and modified the upper with custom-made sock technology, which was thin and made of neoprene material for stretch boots. In the current study, we removed the original shoe uppers and maintained the heel counter and a small part of the toe counter. Neoprene shoe socks (Ultra Stretch Boots, Cressi-Sub, Genoa, Italy) were then glued securely to the sole of each tested shoe (high, medium, and low stiffness). Elliptical holes were prepared at anatomical landmarks to allow direct skin placement of markers with diameters between 25 and 40 mm [28]. All shoe soles of the experimental shoes were painted black to minimize the potential visual impact across the footwear conditions (Figure 1C). Participants were not informed of the actual differences between the shoes.

### 2.3. Procedures

**Figure 2:**
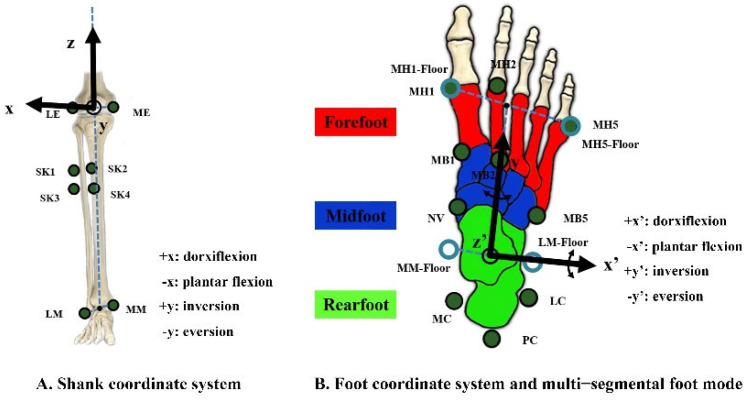
(**A**). A shank coordinate system (xyz) was built to calculate rearfoot-lab and rearfoot-shank kinematics. (**B**). A foot coordinate system (x’y’z’) was built to quantify foot complex motion. Illustration of the marker locations to define the rearfoot (green area), midfoot (dark blue area), and forefoot (red area). Markers at the lateral and medial malleoli (LM and MM), lateral and medial epicondyle of the femur (LE and ME), shank board (SK1-4), first metatarsal head and base (MH1 and MB1), second metatarsal head and base (MH2 and MB2), fifth metatarsal head and base (MH5 and MB5), navicular tuberosity (NV), calcaneus posterior surface (PC), the lateral apex of the peroneal tubercle (LC), and most medial apex of the sustentaculum tali (MC) were attached to the subject.

All reflective markers were placed at marker locations on the skin by the same experienced researcher across all tested conditions and participants (Figure 1C and Figure 2). Eight markers at the lateral and medial malleoli (LM and MM), lateral and medial epicondyle of the femur (LE and ME), and shank board (SK1-4) were added to our marker set to build the shank and foot coordinate systems and track shank movement. The first metatarsal head and base (MH1 and MB1), second metatarsal head and base (MH2 and MB2), fifth metatarsal head and base (MH5 and MB5), navicular tuberosity (NV), calcaneus posterior surface (PC), the lateral apex of the peroneal tubercle (LC), and most medial apex of the sustentaculum tali (MC) were attached over the right foot to define the forefoot, midfoot, and rearfoot segments based on the recommendations suggested by Leardini et al. [29].

According to previous studies [27,30], the bases of the markers were securely attached to their locations throughout the experiment. For each participant, only the balls (spherical markers) were removed from the shoe condition already measured and placed back onto the same bases on the next shoe condition to allow consistent marker locations across the footwear conditions. This arrangement ensured identical marker locations throughout the running trials under all three footwear conditions. The participants’ feet were disinfected before each test, and the neoprene socks were blow-dried after each use to avoid bacterial infection.

A six-camera motion capture system (200 Hz; Vicon, Metrics Ltd., Oxford, UK) was synchronized with a force plate (1000 Hz; OR6GT, 90 × 60 cm, AMTI, Watertown, MA, USA) to collect lower extremity kinematics and GRFs during running. Running trials were performed over a 16 m laboratory runway with a force plate mounted 8 m from the start line. The target running speed was set at 3.3 ± 5% m/s [31] and was controlled by two timing gates (Brower Timing Systems, Salt Lake City, UT, USA), which were placed 3.3 m apart in the middle of the runway. Dominant leg heel strike trials at the target speed with all tracking markers in place and where the entire right foot contacted within the force plate surface during the stance phase were considered successful trials. Practice trials were conducted to familiarize the participants with the test procedure and running speed before data collection. Each participant performed five successful trials for each condition. The order of the shoe conditions was randomized across the participants.

### 2.4. Data Processing

Fourth-order Butterworth low-pass filters with cut-off frequencies of 150 and 30 Hz were applied to the GRF and kinematic data [31,32], respectively. The stance phase was defined from heel contact to toe-off and determined using vertical GRF data at a threshold of 10 N [33]. The entire stance phase was divided into braking and propulsion phases when the time of the anterior-posterior GRF data crossed zero [33,34,35].

A shank coordinate system (xyz) was built to calculate the segmental rotation (rearfoot-lab, rearfoot segment with respect to the laboratory coordinate frame) and joint rotation (rearfoot-shank, rearfoot segment with respect to the shank) kinematics (Figure 2A). The origin was set at the midpoint of the ME and LE, the *z*-axis pointed upward and along the line connecting the origin with the midpoint of MM and LM, and the *x*-axis was orthogonal to an anatomical plane, which was defined among four markers (LM, MM, ME, and LE) using the least squares fit, and the *y*-axis was orthogonal to the x–z plane and followed the right-hand rule. A foot coordinate reference frame (x’y’z’) was built in our study to calculate joint angles, including midfoot-rearfoot (midfoot with respect to the rearfoot), forefoot-rearfoot (forefoot with respect to the rearfoot, a non-adjacent joint) and forefoot-midfoot (forefoot with respect to the midfoot) (Figure 2B). The origin of the reference frame was defined as the midpoint of the two landmarks, which were projected onto the ground plane by MM and ML. The direction of the anterior-posterior (y’) axis joined the origin with the midpoint of two ground-projected landmarks by MH1 and MH5, and the vertical (z’) axis was upward and orthogonal to the transverse plane. The *x*’-axis was orthogonal to the y’–z’ plane, followed by the right-hand rule. All the foot segments share the same foot coordinate system, which allows segments with the same axes of rotation and translation as those of the foot. Specific segmental anatomical tracking markers were defined for each foot segment [29], and the shank was tracked with SK1-4 (Table 1).

Segmental and joint rotations were calculated using Euler angles (sequence sagittal, frontal, and transverse plane motion) according to the International Society of Biomechanics recommendations [36,37], that is, dorsiflexion/plantar flexion as the rotation about the *x*-axis in the sagittal plane, eversion/inversion about the *y*-axis in the frontal plane, and internal/external rotation about the *z*-axis in the transverse plane. During the stance phase, the rearfoot, midfoot, and forefoot kinematics were investigated in the sagittal and frontal planes. Each participant performed a static calibration trial at the neutral standing position before dynamic running trials for each shoe condition, joint angles were defined to be 0° at this position. Dynamic joint angles were calculated relevant to the static calibration trial during the dynamic test. Kinematic data were normalized to 0–100% of the stance phase.

**Figure 3:**
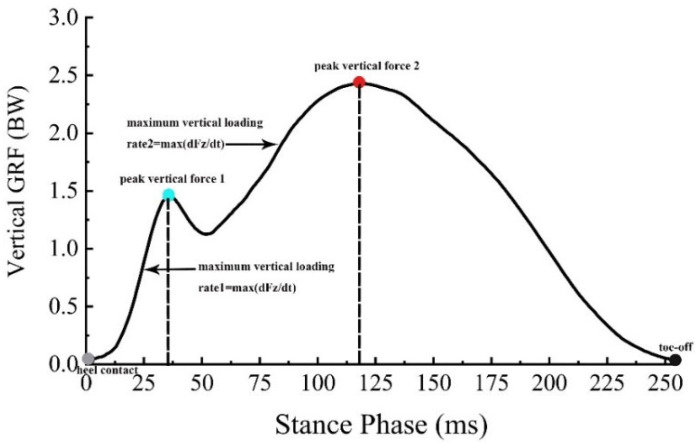
The time profile of vertical ground reaction force (GRF) during the stance phase for one representative trial. The peak vertical force 1 and 2 were shown in blue and red dots, respectively.

The vertical GRF variables included ground contact time, peak vertical force 1, maximum vertical loading rate 1, peak vertical force 2, and maximum vertical loading rate 2 (Figure 3). Ground contact time was defined as the time of the stance phase, which was calculated as the time between heel contact and toe-off. Vertical loading rate 1 was defined as the maximum instantaneous slope of the vertical GRF time profile between the time of heel contact and the time of peak vertical force 1, and vertical loading rate 2 was computed as the maximum instantaneous slope of the vertical GRF time profile between the time of peak vertical force 1 and the time of peak vertical force 2. The extracted anterior-posterior GRF variables were peak braking force, peak propulsion force, braking impulse, and propulsion impulse. The braking and propulsion impulses were calculated as the force-time integral during the braking and propulsion phases, respectively. All force-related variables were normalized to body weight (BW). The kinematic and GRF data from the five trials were averaged for each participant and condition.

### 2.5. Statistical Analysis

Kolmogorov–Smirnov tests were applied to verify that all data were normally distributed. One-way repeated-measures ANOVAs were performed to detect significant differences in shoe midfoot stiffness on the analyzed parameters. When a main effect of the shoe was present, post hoc tests with Bonferroni correction were applied to detect significant differences between every two conditions. Greenhouse–Geisser’s was chosen as an adjustment of the *p*-value when Mauchly’s test of sphericity assumption was rejected. η_p_^2^ was calculated to show the effect size of ANOVA and was categorized as small (0.01 ≤ η_p_^2^ < 0.06), medium (0.06 ≤ η_p_^2^ < 0.14), and large (η_p_^2^ ≥ 0.14) [38]. Statistical significance was set at *p* < 0.05. All statistical analyses were performed using SPSS 26.0 (IBM Corporation, Chicago, IL, USA).

## 3. Results

### 3.1. Kinematics

**Figure 4:**
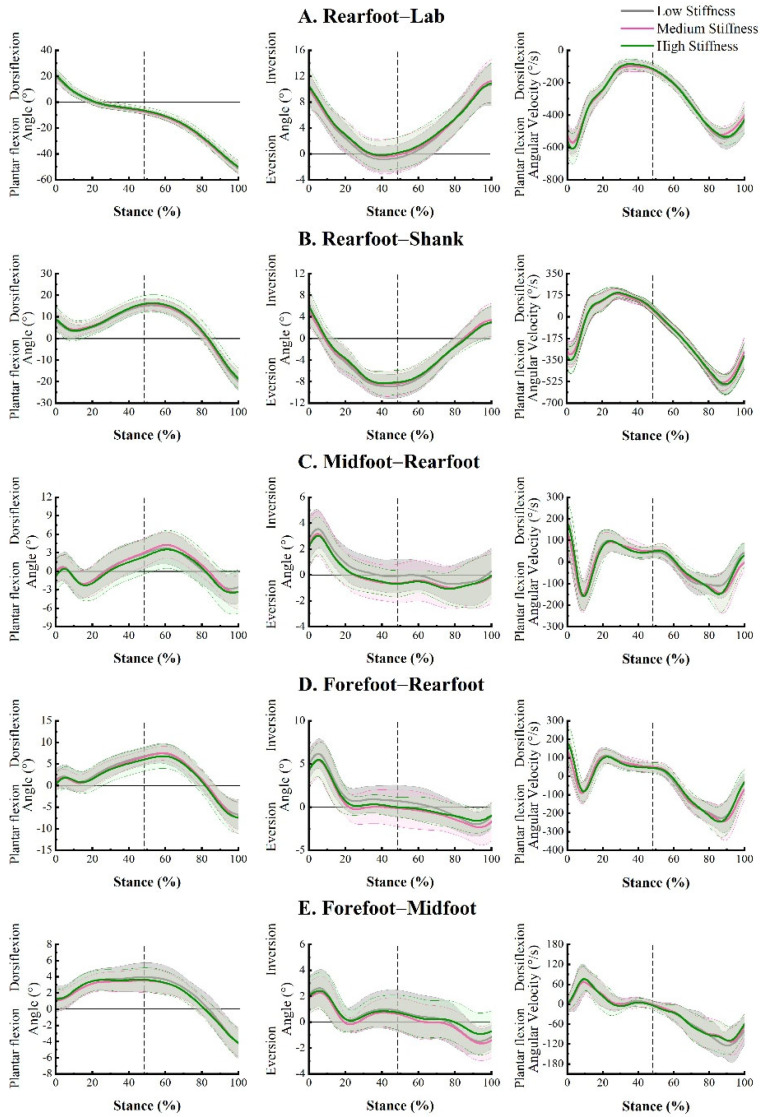
Averaged angle-time curves displayed in the sagittal and frontal planes (first and second columns, respectively) and angular velocity-time curves in the sagittal plane (third column) for rearfoot-lab (**A**), rearfoot-shank (**B**), midfoot-rearfoot (**C**), forefoot-rearfoot (**D**), and forefoot-midfoot (**E**) movements during running stance phase while wearing three shoe conditions. Grey, pink, and green curves separately indicate the low stiffness, medium stiffness, and high stiffness conditions. The dashed line reflects the anterior-posterior force crossed zero.

The multi-segment foot kinematics, including rearfoot-lab, rearfoot-shank, midfoot-rearfoot, forefoot-rearfoot, and forefoot-midfoot interactions of the shoe conditions are displayed in Figure 4. Angle curves in the sagittal and frontal planes and angular velocity curves in the sagittal plane were averaged for all participants and normalized to 0–100% during the stance phase. The 0° angle outlined in the sagittal and frontal planes indicated that the angle of the joint was consistent with the static calibration trial. The rearfoot-lab, rearfoot-shank, midfoot-rearfoot, forefoot-rearfoot, and forefoot-midfoot kinematic variables are provided in Table 2.

For rearfoot-lab interactions in the sagittal plane, the dorsiflexion angle at initial contact (F (2, 36) = 3.270, *p* = 0.050, η_p_^2^ = 0.154) did not show any significant difference. A significant main effect of the shoe was observed for peak plantar flexion angular velocity during the braking phase (F (2, 36) = 3.697, *p* = 0.035, η_p_^2^= 0.170). However, Bonferroni post hoc comparisons did not find any difference between shoe conditions. No significant difference was found in rearfoot-shank peak eversion angle and frontal plane range of motion between shoe conditions (*p* > 0.05).

None of the midfoot-rearfoot variables, including the peak dorsiflexion angle, frontal plane range of motion, and peak plantar flexion angular velocities, showed any differences in sagittal and frontal plane movements between shoes during braking and propulsion phases (*p* > 0.05).

A significant main effect (F (2, 36) = 6.247, *p* = 0.005, η_p_^2^ = 0.258) was observed in the frontal range of motion of the forefoot-rearfoot joint. The magnitude was greater in the low stiffness shoe (8.7 ± 2.1°) than that in the high stiffness shoe (7.8 ± 2.0°) during the stance phase.

The forefoot-midfoot variable indicated a significantly greater frontal plane range of motion (F (2, 36) = 4.399, *p* = 0.020, η_p_^2^ = 0.196) in the medium stiffness shoe (4.6 ± 0.9°) than in the high stiffness shoe (4.2 ± 1.1°). A significant main effect (F (1.430, 25.747) = 4.009, *p* = 0.043, η_p_^2^ = 0.182) of the shoe condition was found when comparing the peak plantar flexion velocity during the propulsion phase, and no significant difference was observed for post hoc comparisons.

### 3.2. GRF

**Figure 5:**
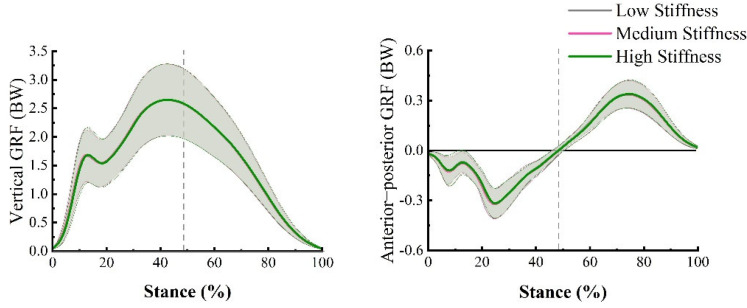
Averaged force-time curves displayed the vertical and anterior-posterior GRF. Grey, pink, and green curves separately indicate the low stiffness, medium stiffness, and high stiffness conditions. The dashed line reflects the anterior-posterior force crossed zero.

The averaged vertical and anterior-posterior GRF curves are displayed in Figure 5, which show very similar patterns across shoe conditions during the stance phase of running. No significant differences were found in the ground contact time, peak vertical force 1, maximum vertical loading rate 1, peak vertical force 2, maximum vertical loading rate 2, peak braking force, peak propulsion force, braking impulse, and propulsion impulse between shoe conditions (*p* > 0.05; Table 3).

## 4. Discussion

This study examined the influence of shoe midfoot stiffness on multi-segment foot movements and GRF during heel-toe running. The results of this study provide insights into the underlying mechanism of footwear midfoot stiffness on foot joint kinematics and GRF characteristics. Our study showed similar patterns of foot segmental motion across shoe conditions during the stance phase of running (Figure 4). However, the results of this study indicate that changes in midfoot stiffness can influence foot motion between high stiffness shoes and low stiffness or medium stiffness shoes. Our present study found no significant differences between low stiffness and medium stiffness conditions for the intersegmental foot kinematics in both the sagittal and frontal planes, which may be due to the small mechanical property differences between shoes.

Our study’s initial rearfoot-lab dorsiflexion angle >8.0° confirmed that all participants performed rearfoot strike patterns [39]. The influence of shoe midfoot stiffness on the initial rearfoot-lab dorsiflexion angle did not show any difference. This is in line with a previous study on shoe forefoot hardness, which showed the same sagittal shoe-ground angle at the initial ground contact [31]. These results may be explained by the fact that the rearfoot structure and material were identical for the low, medium, and high midfoot stiffness shoe conditions, resulting in similar ground contact kinematics.

During the propulsion phase, the participants displayed peak midfoot-rearfoot, forefoot-rearfoot, and forefoot-midfoot plantar flexion velocities. It is assumed that the increased midfoot bending stiffness could increase the resistance in the midfoot region, which could decrease the plantar flexion speed during the propulsion phase. Previous studies also found that an increase in shoe stiffness decreases the maximum MTP plantar flexion [17] and peak arch flexion velocities [10] during the terminal stance. Our results indicated that midfoot bending stiffness had a significant main effect on forefoot-midfoot peak plantar flexion angular velocity during the propulsion phase. Still, no significant difference could be determined between shoe conditions. Larger effects may be possible if greater modifications are made to the experimental shoes.

Increased shoe forefoot [11] and full-length [10] midsole bending stiffness reduced arch deformation during walking and running, respectively. However, none of the midfoot-rearfoot, forefoot-rearfoot, and forefoot-midfoot peak dorsiflexion angles showed any significant difference between shoe conditions in our study. The results of the current study suggest that alterations in midfoot stiffness would not be sufficient to vary the amount of midfoot deformation. This may be explained by the fact that the midfoot plate was too short to generate a lever arm effect on arch movement.

Participants wearing shoes with higher midfoot bending stiffness demonstrated smaller forefoot-midfoot and forefoot–rearfoot frontal plane range of motion during the running stance phase. Still, no significant differences were found for the midfoot-rearfoot joint. Renan et al. [11] found that shoes with drilled holes in the forefoot increased the forefoot-rearfoot inversion angle in the frontal plane. However, Arndt et al. [15] showed no increase in frontal plane ranges of motion when walking with shoes with lower midfoot bending stiffness. The differences across studies may be due to the small number of participants or insufficient cuts to create a softer stiffness prototype in their study and the higher exercise intensity of the participants in our study. The forefoot-rearfoot motion in the frontal plane is considered a foot torsional motion which can be decreased when the shoe torsional stiffness increases [13,14]. Although we did not quantify the torsional stiffness of the shoe conditions, the decreased torsional movement may be explained by the increased shoe torsional stiffness under the high bending stiffness condition.

Previous studies indicated that increased foot torsional stiffness restricts the forefoot-rearfoot motion in the frontal plane, and thus leads to an excessive motion of the ankle pronation [12,14], which is considered a risk factor for running-related injuries [40,41,42]. Stacoff et al. found that running with a shoe decreases the torsion movement of the foot, and increases the pronation of the foot compared with barefoot running [12]. Another study by Graf et al. observed a larger ankle range of motion with increasing shoe torsional stiffness [14], whereas Graf and Stefanyshyn [13] did not find any difference in ankle kinematics for shoes with different torsion ranges of motion. In our study, midfoot stiffness did not affect rearfoot-shank motion in the frontal plane, suggesting that the increase in midfoot stiffness alone may have minimal effect on pronation-related injuries.

No significant differences were found in the peak vertical forces and instantaneous loading rates between the shoe conditions. Oh and Park [18] also found that shoes with carbon fiber insoles did not influence vertical GRF data, which indicates that the change in shoe bending stiffness at the midfoot area would not influence the vertical GRF. Moreover, Oh and Park [18] investigated a longer stance duration and a smaller anterior-posterior GRF when wearing shoes with carbon fiber insoles, but no significant difference was found for the anterior-posterior GRF impulse. In our study, the ground contact time, peak anterior-posterior forces, and the anterior-posterior impulses did not differ among shoe conditions. The participants maintained their anterior-posterior impulses to keep the same running velocity across the different shoes.

Some limitations of this study must be acknowledged when interpreting the results. First, the shoe upper was mostly modified, which may have sacrificed the original function of the shoe support (e.g., shoe upper stability). However, the elastic sock upper is a good alternative to bone-pin markers for the non-invasive measurement of actual foot multi-segment. Second, only laboratory ground running was measured in the present study and studying midfoot stiffness during long-distance running should be considered. Third, although our primary scope of study aimed to analyze the actual multi-segment foot kinematics, running shoe midfoot stiffness might influence the other proximal joint kinematics (ankle, knee, or hip) according to the kinetic chain theory. Fourth, this study did not analyze the multi-segment foot kinetic influences of running shoe midfoot stiffness as no reliable anthropometric data are available, such as the center of mass, for accurate foot segment calculation. Fifth, only males were recruited in our study as the tested shoes were built in US size 9, and the current results may not be generalized to females. Finally, we just tested three shoe conditions whose stiffness are 0.069, 0.091, and 0.163 Nm/°, and future research should consider a larger range of midfoot stiffness conditions to optimize the midfoot stiffness in the running community.

## 5. Conclusions

The midfoot stiffness of running shoes can influence multi-segment foot movements during heel-toe running. Shoes with increased midfoot bending stiffness can reduce foot medial-lateral movements during the stance phase of running, indicating that the length variation of intersegmental muscles and tendons should also be smaller, which may decrease the tension of the foot muscles and tendons connected between the metatarsals and tarsus. However, we found no difference between shoe conditions in ankle kinematics, thus the effect of shoe midfoot stiffness related to pronation injury was not evident.

## Figures and Tables

**Table 1:** Tracking markers for foot and shank segments.

Segment	Tracking Markers
Rearfoot	PC, LC, and MC
Midfoot	MB2, MB5, and NV
Forefoot	MH1, MH2, MH5, MB1, and MB5
Shank	SK1-4

**Table 2:** Multi-segment kinematic variables (Mean ± SD [95% confidence interval]) of experimental shoes. A significant main effect *p*-value is bolded.

Segment Interaction	Variable	Low Stiffness	Medium Stiffness	High Stiffness	*p*-Value	η_p_^2^
Rearfoot-lab	Dorsiflexion angle at initial contact (°)	19.6 ± 4.1 [17.6, 21.6]	20.5 ± 4.9 [18.1, 22.8]	21.1 ± 4.8 [18.8, 23.4]	0.050	0.154
	Peak plantar flexion angular velocity during the braking phase (°/s)	577.4 ± 73.5 [542.0, 612.8]	580.5 ± 81.5 [541.2, 619.8]	617.4 ± 96.0 [571.1, 663.6]	**0.035**	0.170
Rearfoot-shank	Peak eversion angle (°)	9.1 ± 2.2 [8.0, 10.1]	8.7 ± 2.2 [7.6, 9.8]	−8.5 ± 2.2 [7.5, 9.6]	0.360	0.055
	Frontal plane range of motion (°)	14.2 ± 3.1 [12.7, 15.6]	14.0 ± 4.0 [12.0, 15.9]	14.5 ± 3.9 [12.6, 16.4]	0.389	0.051
Midfoot-rearfoot	Peak dorsiflexion angle (°)	4.1 ± 2.4 [2.9, 5.2]	4.5 ± 2.1 [3.5, 5.5]	3.9 ± 2.8 [2.6, 5.3]	0.630	0.025
	Frontal plane range of motion (°)	5.3 ± 2.0 [4.3, 6.2]	5.2 ± 1.9 [4.3, 6.0]	5.1 ± 2.0 [4.2, 6.1]	0.812	0.012
	Peak plantar flexion angular velocity during the braking phase (°/s)	188.5 ± 37.7 [170.3, 206.6]	176.2 ± 64.4 [145.1, 207.2]	182.1 ± 51.5 [157.2, 206.9]	0.653	0.023
	Peak plantar flexion angular velocity during the propulsion phase (°/s)	151.7 ± 41.2 [131.8, 171.5]	177.3 ± 62.7 [147.1, 207.5]	172.1 ± 70.2 [138.2, 205.9]	0.080	0.143
Forefoot-rearfoot	Peak dorsiflexion angle (°)	7.7 ± 2.3 [6.6, 8.8]	7.7 ± 1.6 [7.0, 8.5]	7.0 ± 2.7 [5.7, 8.3]	0.428	0.046
	Frontal plane range of motion (°)	8.7 ± 2.1 [7.7, 9.7]	8.4 ± 2.0 [7.4, 9.4]	7.8 ± 2.0 [6.8, 8.8]	**0.005 ^a^**	0.258
	Peak plantar flexion angular velocity during the braking phase (°/s)	109.8 ± 40.3 [90.4, 129.3]	106.2 ± 46.2 [83.9, 128.5]	100.8 ± 47.5 [77.9, 123.7]	0.680	0.021
	Peak plantar flexion angular velocity during the propulsion phase (°/s)	244.9 ± 55.7 [218.0, 271.7]	266.1 ± 77.6 [228.7, 303.6]	265.5 ± 63.3 [235.0, 296.0]	0.066	0.140
Forefoot-midfoot	Peak dorsiflexion angle (°)	4.4 ± 1.7 [3.5, 5.2]	3.9 ± 1.4 [3.3, 4.6]	4.3 ± 1.3 [3.6, 4.9]	0.135	0.105
	Frontal plane range of motion (°)	4.7 ± 1.3 [4.1, 5.4]	4.6 ± 0.9 [4.2, 5.0]	4.2 ± 1.1 [3.7, 4.8]	**0.020 ^b^**	0.196
	Peak dorsiflexion angular velocity during the braking phase (°/s)	102.0 ± 33.5 [85.9, 118.2]	95.2 ± 32.0 [79.8, 110.6]	102.5 ± 34.0 [86.1, 118.9]	0.512	0.036
	Peak plantar flexion angular velocity during the propulsion phase (°/s)	154.4 ± 46.0 [132.2, 176.6]	144.8 ± 35.8 [127.6, 162.0]	136.5 ± 32.7 [120.7, 152.2]	**0.043**	0.182

^a^ significant difference in low stiffness and high stiffness conditions. ^b^ significant difference in medium stiffness and high stiffness conditions.

**Table 3:** GRF-related variables (Mean ± SD [95% confidence interval]) of experimental shoes.

Variable	Low Stiffness	Medium Stiffness	High Stiffness	*p*-Value	η_p_^2^
Ground contact time (ms)	232.8 ± 17.3 [224.5, 241.1]	233.4 ± 15.9 [225.8, 241.1]	233.4 ± 16.2 [225.6, 241.2]	0.870	0.008
Peak vertical force 1 (BW)	1.74 ± 0.27 [1.61, 1.87]	1.75 ± 0.30 [1.61, 1.90]	1.75 ± 0.31 [1.60, 1.90]	0.858	0.008
Maximum vertical loading rate 1 (BW/s)	105.9 ± 25.2 [93.8, 118.1]	109.9 ± 29.5 [95.7, 124.1]	107.8 ± 27.61 [94.5, 121.1]	0.941	0.003
Peak vertical force 2 (BW)	2.67 ± 0.22 [2.56, 2.77]	2.67 ± 0.21 [2.60, 2.77]	2.67 ± 0.22 [2.57, 2.78]	0.336	0.059
Maximum vertical loading rate 2 (BW/s)	39.58 ± 6.69 [36.36, 42.81]	39.00 ± 5.84 [36.19, 41.82]	39.37 ± 6.96 [36.01, 42.72]	0.743	0.016
Peak braking force (BW)	0.34 ± 0.01 [0.33, 0.36]	0.34 ± 0.01 [0.33, 0.36]	0.35 ± 0.01 [0.33, 0.36]	0.886	0.008
Peak propulsion force (BW)	0.35 ± 0.01 [0.32, 0.38]	0.35 ± 0.01 [0.32, 0.37]	0.34 ± 0.01 [0.31, 0.37]	0.340	0.058
Braking impulse (Ns/BW)	0.016 ± 0.003 [0.018, 0.014]	0.016 ± 0.003 [0.018, 0.015]	0.016 ± 0.004 [0.018, 0.014]	0.574	0.030
Propulsion impulse (Ns/BW)	0.023 ± 0.003 [0.021, 0.024]	0.023 ± 0.003 [0.021, 0.024]	0.023 ± 0.003 [0.021, 0.024]	0.886	0.007

## Data Availability

The data presented in this study are available on request from the corresponding author.
